# Applying reflective multicriteria decision analysis to understand the value of therapeutic alternatives in the management of gestational and peripartum anaemia in Spain

**DOI:** 10.1186/s12884-022-04481-w

**Published:** 2022-02-25

**Authors:** Manel Casellas Caro, María Jesús Cancelo Hidalgo, José Antonio García-Erce, José Luis Baquero Úbeda, Maria Glòria Torras Boatella, Elena Gredilla Díaz, Margarita Ruano Encinar, Israel Martín Bayón, Jordi Nicolás Picó, José Eduardo Arjona Berral, Alberto Muñoz Solano, Silvia Jiménez Merino, Mónica Cerezales, Jesús Cuervo

**Affiliations:** 1 Department of Obstetrics, Hospital Universitari Vall d´Hebron, Passeig de la Vall d’Hebron, 119, 08035 Barcelona, Spain; 2 Department of Obstetrics and Gynecology, Hospital Universitario Guadalajara, Calle Donante de Sangre, 19002 Guadalajara, S/N Spain; 3 Banco de Sangre Y Tejidos de Navarra, Servicio Navarro de Salud-Osasunbidea, Calle Irunlarrea, 3, 31008 Pamplona, Spain; 4 Grupo Español de Rehabilitación Multimodal (GERM), Instituto Aragonés de Ciencias de La Salud, Avenida San Juan Bosco, 13, 50009 Zaragoza, Spain; 5 PBM Group, Hospital La Paz Institute for Health Research (IdiPAZ), Paseo de la Castellana, 261, 28046 Madrid, Spain; 6 Foro Español de Pacientes, Calle Volver a Empezar, 4 portal H, 1 B, 28018 Madrid, Spain; 7 Àrea d’Innovació, Hospital Universitari Bellvitge, Carrer de La Feixa Llarga, L’Hospitalet de Llobregat, 08907 Barcelona, S/N Spain; 8 Institut Català de La Salut, Barcelona, Spain; 9 Anaesthesia Department, Hospital La Paz, Paseo de La Castellana, 261, 28046 Madrid, Spain; 10 Hospital Pharmacy Department, Hospital La Paz, Madrid, Spain; 11 CS Polop-La Nucía, Avenida de Sagi Barba, 24, Polop, La Nucía, 03520 Alicante, Spain; 12 Hospital Universitari Mutua Terrasa, Plaça del Doctor Robert, 5, 08221 Terrassa, Spain; 13 Hospital San Juan de Dios, Avenida del Brillante 106, 14012 Córdoba, Spain; 14 Department of Obstetrics and Gynecology, Hospital Universitario Marqués de Valdecilla, Avenida de Valdecilla, 25, 39008 Santander, Spain; 15 Vifor Pharma España S.L.U., Avinguda Diagonal, 611, 08028 Barcelona, Spain; 16 Axentiva Solutions S.L., Calle Monte Cerrau, 28, 33006 Asturias, Oviedo Spain

**Keywords:** Technology assessment, Anemia, Pregnancy, Clinical decision-making, Shared decision making, Ferric carboxymaltose

## Abstract

**Background:**

The objective of the FeminFER project was to assess the value of ferric carboxymaltose following a multicriteria decision analysis in obstetrics and gynaecology in Spain.

**Methods:**

Ferric carboxymaltose (FCM) and ferrous sulphate were evaluated using the EVIDEM framework. Ten stakeholders participated to collect different perspectives. The framework was adapted considering evidence retrieved with a PICO-S search strategy and grey literature. Criteria/subcriteria were weighted by level of relevance and an evidence-based decision-making exercise was developed in each criterion; weights and scores were combined to obtain the value of intervention relative to each criterion/subcriterion, that were further combined into the Modulated Relative Benefit-Risk Balance (MRBRB).

**Results:**

The most important criterion favouring FCM was Compared Efficacy/Effectiveness (0.183 ± 0.07), followed by Patient Preferences (0.059 ± 0.10). Only Direct medical costs criterion favoured FS (-0.003 ± 0.03). MRBRB favoured FCM; 0.45 ± 0.19; in a scale from -1 to + 1.

**Conclusions:**

In conclusion, considering the several criteria involved in the decision-making process, participants agreed with the use of FCM according to its MRBRB.

**Supplementary Information:**

The online version contains supplementary material available at 10.1186/s12884-022-04481-w.

## Background

Iron deficiency anaemia (IDA) is the most prevalent nutritional deficiency worldwide, being present in 29% non-pregnant women, 38% pregnant women, and more than 40% of children [1]. Data for the prevalence of anaemia in Europe reports rates of 11% (6–20) for children under 5 years old, 16% (12–22) for non-pregnant women aged 15–49, and 22% (16–29) for pregnant women between 15–49 years old [2], this data is in accordance with a national study reporting a prevalence of 22.6% of anaemia in pregnant women aged 16–43 [3]. Women are specially affected by IDA due to blood loss caused by menstruation. During pregnancy, anaemia is common due to higher requirements of iron and the effect of haemodilution caused by an increase in plasma volume [4]. IDA during pregnancy has been reported to be associated with intra- and postpartum haemorrhage and transfusion need [5] and it is not just the mother who is affected by IDA but the new-born as well, as iron deficiency affects growth and organ functioning, the immune system is also altered, and neurodevelopmental impairments and predisposition to postnatal iron deficiency have been reported [6].

Routine clinical care for these patients has been oral iron, normally ferrous sulphate (FS), and intravenous (IV) iron in those cases of severe IDA or newly diagnosed pregnant women beyond 34 weeks of pregnancy because of its rapid effectiveness in rising iron levels to correct the anaemia before labour as it is likely to result in excessive bleeding, post-labour anaemia, and use of blood products such as blood transfusion [7, 8]. In Spain, a guide on the management of IDA in pregnancy, follows this same approach of using oral iron when possible and IV iron for those moderate-severe, non-responder, or last minute cases [9]. Nevertheless, a study carried out in a Spanish hospital about the use of IV iron reported that 24.82% of the patients requiring this treatment were related to the obstetric department [10].

Ferric carboxymaltose (FCM) was approved in Spain in 2008 and by the European Medicines Agency in 2007 and its labelled indications are: oral iron non-responder patients, when oral iron cannot be used, and when there is an urgent need for iron. Specifically for pregnant women, FCM can be only administered in the second or third trimesters of pregnancy and even though its clinical benefits above oral iron have been demonstrated in several clinical trials [11–19] it is not widely used, what seems to be driven by its higher acquisition cost [20, 21].

With this background and these especially sensitive patients it seems adequate to evaluate the factors that might have an influence and that are relevant for the different stakeholders involved in the decision-making process, from a holistic perspective. Currently the incorporation of patient preferences and the patients’ perspective is a discussion point and decision-making processes are moving forward to the implementation of this approaches [22]. Multicriteria Decision Analysis (MCDA) through the EVIDEM framework, enables the inclusion of a comprehensive group of criteria that are relevant for establishing the value of the intervention in a specific health system in an explicit, replicable, and systematic way.

The Spanish Patient Forum (Foro español de pacientes, https://forodepacientes.org/) has promoted the FeminFER project which is also supported by the Spanish Society of Gynaecology and Obstetrics (SEGO, https://sego.es/) and the Spanish Society for Healthcare Quality (SECA, https://calidadasistencial.es/) with the objective to assess the value of interventions, FS and an intravenous agent -FCM-, targeting gestational and peripartum anaemia by means of a comprehensive, reflective approach involving all the partners taking part in the clinical situation: gynaecologists/obstetricians, haematologists, anaesthesiologists, midwives, hospital pharmacists, decision-makers, and patients and patients’ representative in Spain.

## Methods

The study was designed following the EVIDEM framework [23], which includes predefined domains and criteria in a core model (quantitative) and contextual criteria (qualitative) that were further specified with additional subcriteria relating to the specific disease. This EVIDEM framework has been widely used internationally [24, 25] and specifically in Spain [26], for example it has been recently validated to complement the decision-making process for orphan medicines [27]. The treatments subjected to evaluation were FCM (IV treatment) and ferrous sulphate, FS, (oral treatment) according to the usual care as defined by the clinical guidelines [7, 8, 28]. To establish the subcriteria and collect the published evidence for each of them, a literature review was conducted. All the information was structured and organized to allow the decision-makers to choose in two exercises: hierarchical point allocation method and direct rating scale method. A multidisciplinary panel of Spanish stakeholders constituted the decision-making group and scored the evidence.

### Literature review

A systematic literature review (SLR) was conducted with two purposes, the first one was to define the specific subcriteria within the framework for the indication, using the EVIDEM domain-criteria framework, further subcriteria were identified according to the SLR identifying all the relevant characteristics to be considered in the decision-making process such as efficacy, safety, patient preferences, economic evaluations, political aspects, and others. The second objective of the SLR was to create the evidence matrix for each criterion and subcriterion. A medical database targeted search following the PICO approach (*population, intervention, comparison, and outcomes*) [29] was carried out, including a time horizon (2010-present) and type of studies (randomized clinical trials, systematic reviews, meta-analysis, observational studies) limitation; databases used were PubMed and Cochrane Library (Additional Figure 1, Additional Tables 1 and 2). Additional documents and evidences were searched in the grey literature, for example clinical practice guidelines, clinical trials [30], the European Medicines Agency [31], Agencia Española de Medicamentos y Productos Sanitarios [32], Anemia Working Group España [33], and Network for the Advancement of Patient Blood Management, Haemostasis and Thrombosis [34].

### MCDA evidence matrices

**Fig. 1:**
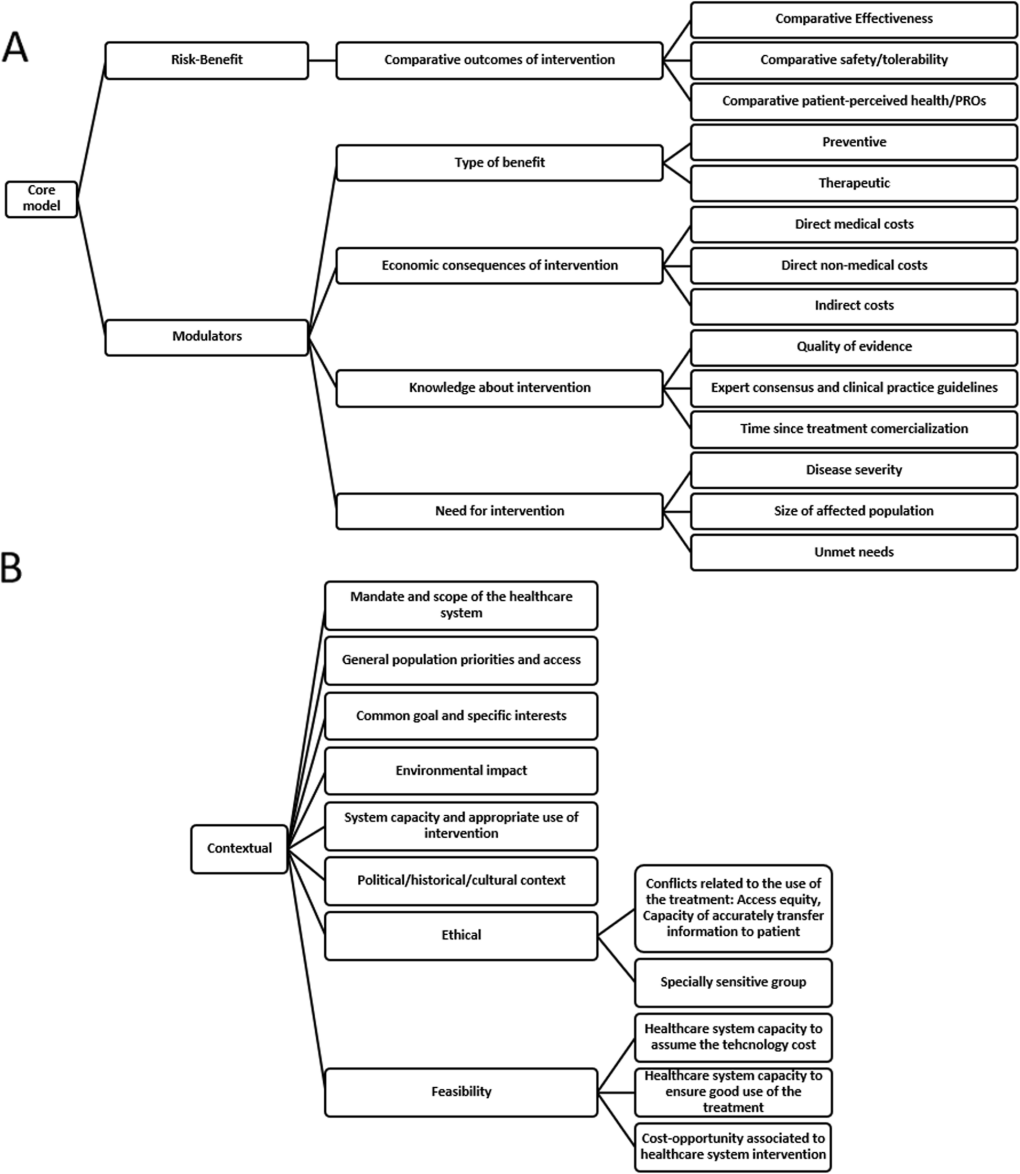
Domains, criteria, and subcriteria selected for the evaluation of health technologies in the management of pregnancy anaemia and anaemia in the peripartum period in Spain; 1A, Core model, 1B, contextual criteria

The EVIDEM framework was used to develop the evidence matrices, the framework includes the core model (risk–benefit, and modulators domains), that is further itemised in general and specific crit﻿eria﻿﻿ (Fig. 1a), and the contextual criteria (Fig. 1b). Additional contextual criteria were included in the EVIDEM framework according to the report published by OSTEBA, the Agency of evaluation of Health Technologies of the Basque Country [35].

### Expert panel, workshops, and exercises

An expert panel composed of twelve people was created to collect insights from the different perspectives in the management of anaemia in gynaecology. There were clinical experts including the following profiles which are involved within the context of the gynaecological indication of interest gynaecology/obstetrics, midwifery, haematology, anaesthesiology, hospital pharmacy, regional decision makers, patient representatives, and patients. Due to national travelling restrictions, lockdowns, and public health situation all the exercises and meetings were carried out in a virtual approach developing online tools that fulfilled all the needs.

In a first basis all the experts received documents with the methodology of the project. The first phase of the project was selecting the specific subcriteria for the indication, that were validated as clinically accurate by the principal investigator of the project and to ensure their understandability, definitions were reviewed and validated by the patient involved. These criteria and subcriteria were subjected to Hierarchical Point Allocation method by the experts in a virtual workshop in order to obtain those aspects that were more relevant in the Spanish context when making a decision; this was performed by distributing 100 weighting points across every set of domains, criteria, and subcriteria; this rating method was preferred over the direct rating method also proposed by EVIDEM, as it forces the experts to weight each criterion/subcriterion while keeping in mind the rest of the criteria/subcriteria within the same level; this approach forces the prioritization of criteria/subcriteria and not allowing the participants to state all of them as very relevant or not relevant at all. After that, all weights were normalized. In the second phase of the project a description of the criteria/subcriteria based on the available evidence was presented to the participants and they reflected how the evidence on the criterion would impact their decision by using a direct rating scale method, in which they by assigned a score from + 5 (“Much in favour of option a”) to -5 (“Much in favour of option b”). For the contextual criteria, participants selected the type of the impact in their decisions of these criteria:”In favour of FS”, neutral, or”In favour of FCM”.

Two online tools were developed for the two exercises with the purpose of simplifying the participation and collection of data.

A final virtual workshop was carried out to share and reflect individual preferences on the value of compared alternatives, to discuss final results and to include any additional individual comments, this phase was of great importance to the project as it is the part in which the different participants’ perspectives are shared in order to reach an agreement and establish meeting points to improve patients’ management.

### Data analysis

In the first exercise to define the conceptual framework by using hierarchical point allocation, the relative weight of the criteria was investigated; results were exported to an excel file, their relative weight was estimated taking into account the hierarchical model, normalised (*Wx*), and converted to a 0–1 scale by using a lineal scale, in which 0 meant “no weight” and 1 “maximum weight”.

After the second exercise, direct rating scale, the mean scores were standardized in a scale from -1 to + 1 (*Sx*). Lastly, the value of intervention (*Vi*) of each criterion or subcriterion was calculated as the product of their normalised weight (from the first exercise, *Wx*) and the standardised score of the second exercise (*Sx*). These results were presented as mean ± standard deviation (SD) and represented in graphs. The final modulated relative benefit-risk balance (MRBRB) was calculated as the sum of the Vi of all subcriteria (*x*), in an additive linear model.

$$MRBRB=\sum_{x=1}^{n}Vi= \sum_{x=1}^{n}{W}_{x} x {S}_{x}$$

The contextual criteria were represented in a qualitative scale with the three options: positive, neutral, or negative impact.

## Results

According to the search strategy, 29 studies were chosen to be included (Additional Figure 2), however additional evidence was searched ad hoc when needed.

There were twelve participants in the decision-making process: four gynaecologist/obstetric specialists, one haematologist, one anaesthesiologist, one midwife, two hospital pharmacists, a healthcare system decision-maker, a patient, and a patient’s representative.

### Adapting the EVIDEM Framework for a benign gynaecological condition: criteria weights

**Fig. 2:**
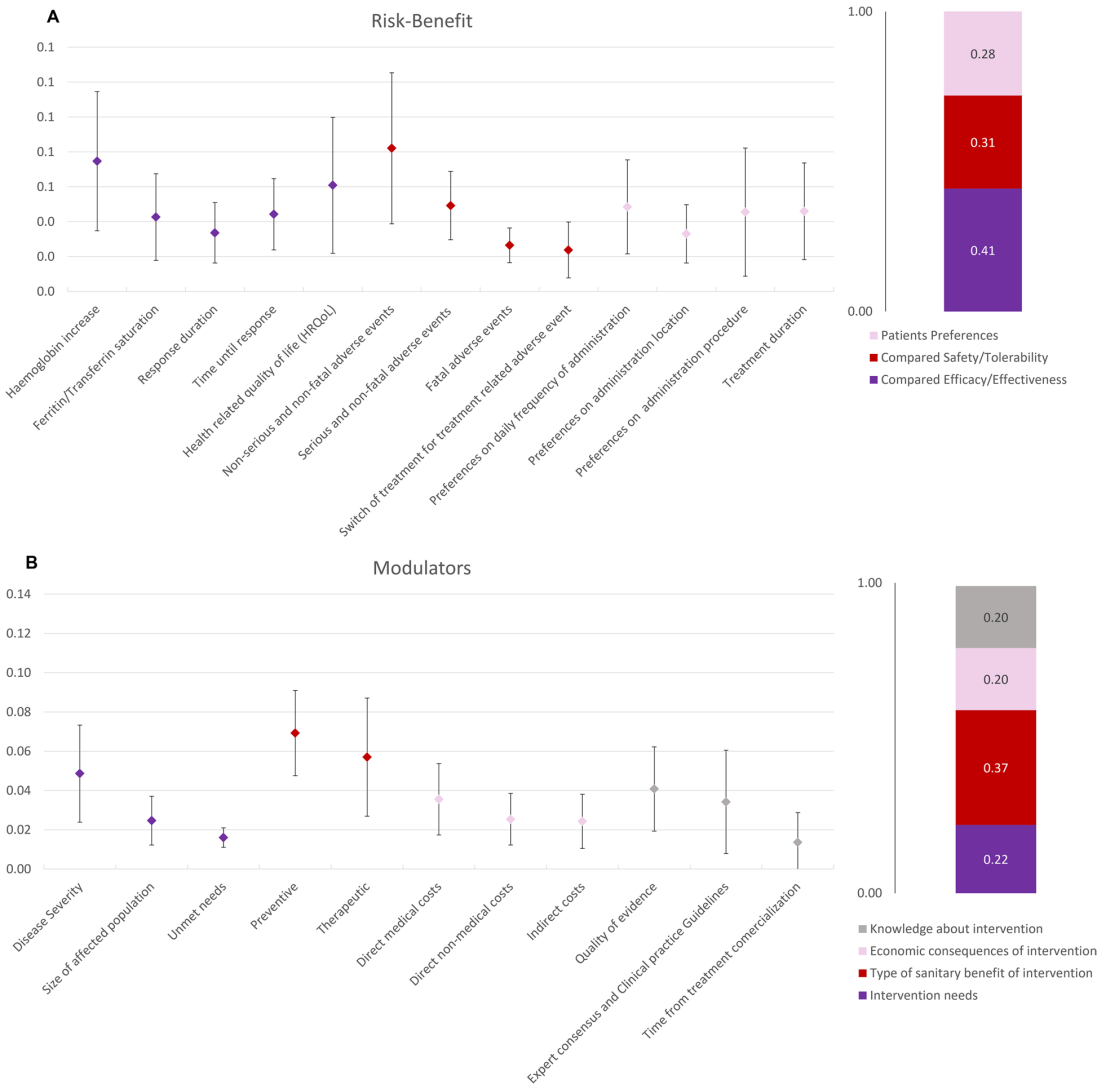
Mean and standard deviations of the normalised weights assigned to criteria and subcriteria by hierarchical point allocation in the core model (risk–benefit, 2A, and modulators, 2B)

Using hierarchical point allocation method to the core model, weights resulted in 62% (± 8%) risk–benefit criteria, and 0.38 (± 0.08) for the modulators (Fig. 2). Within risk–benefit domain, compared efficacy-effectiveness criterion received the greatest weight, 0.26 (± 0.10), with values ranging 0.06–0.48 across the different professional profile of the participants, corresponding subcriteria ranged between 0.03 (± 0.02) (Response duration) and 0.08 (± 0.06) (Haemoglobin increase). Compared safety/tolerability was weighted as 0.19 (± 0.06) of the risk–benefit, and the most important subcriterion was Non-serious and non-fatal adverse events (0.09 (± 0.05)), followed by Serious and non-fatal adverse events (0.05 (± 0.02)). The last criterion within the Risk–Benefit domain, Patient’s preferences, received a weight of 0.17 (± 0.11), being the most important Preferences on daily frequency of administration, 0.05 (± 0.05).

As additional contextual criteria were selected according to Spanish agency recommendations, these criteria were also subjected to hierarchical point allocation method to assess their appropriateness. Those criteria receiving higher weights were Feasibility (0.21 ± 0.12), General population priorities and access (0.20 ± 0.10), Ethical (0.20 ± 0.18), and Mandate and scope of the healthcare system (0.19 ± 0.09). The remaining criteria received very low weights and were excluded for being non-relevant: Common goals and specific interests (0.06 ± 0.06), Environmental impact (0.09 ± 0.07), and Political/historical/cultural context (0.04 ± 0.05).

### Criteria weights by profile

Within de Risk–Benefit domain, all the profiles were aligned with valuations around 60% (minimum 50% for the healthcare decision-maker profile, and maximum 70% for the hospital pharmacists), in general the most important criterion was Compared Efficacy/Effectiveness (ranging 15%-36%), while anaesthesiologists and healthcare system decision-makers considered equally important Compared Safety/Tolerability (27% and 15% each, respectively). Additionally, for the healthcare system decision-makers and patients/patients’ representatives the most important criterion within the Risk–Benefit domain was Patient preferences (20% for the decision-makers, and 33.4% for the patients and patients’ representatives). Those giving more importance to Compared Safety/Tolerability were anaesthesiologists and hospital pharmacists (24%), while patients/patients representatives were valuating it the least (12.4%). Patients’ preferences were particularly important for patients/patients’ representatives, followed by haematologists (24%), those giving less importance to this criterion were anaesthesiologists (6%).

Modulators were as important as the Risk–Benefit domain for the decision-makers (50%-50%), but less important for the other profiles (30%-40%). The Type of benefit of the intervention was valuated as the most important criterion or as important as the other criteria by all the participants; preventive benefit was more important than therapeutic benefit for some profiles; anaesthesiology, haematology, decision-makers, and patients/patients’ representatives; while hospital pharmacy, and gynaecology/obstetrics considered the therapeutic benefit as more relevant; midwifery considered both criteria as equally relevant (10% each).

Regarding the economic consequences of the intervention, decision-makers were the group valuating the most this criterion (15%) and its subcriteria, followed by anaesthesiology (10%), and haematology (10%). These groups were also giving more importance to the Knowledge about intervention (20% decision-makers, and 10% anaesthesiology and haematology), while the rest of the participants did not consider this criterion as truly relevant (3–8.5%).

### Defining the value of compared interventions for gestational and peripartum anaemia: Decision-making

Participants chose between FCM or FS in relation to each criterion and subcriterion basing the decision on available evidence (Table 1). All participants were much in favour of FCM regarding Compared efficacy/effectiveness (3.52 ± 1.08) and most of its subcriteria, whilst Compared safety/Tolerability was slightly in favour of FCM (1.16 ± 2.62), but some participants chose FS; when considering Patients Preferences (1.95 ± 2.64); Daily frequency of administration (3.36 ± 2.06) and Duration of treatment (3.88 ± 2.60) seemed to be driving the decision towards FCM, while Preferences on administration location (0.48 ± 3.53) and administration procedure (-0.48 ± 3.13) were more neutral or in favour of FS.

**Table 1:** Mean and standard deviations (SD) of the decision-making exercise of iron carboxymaltose versus ferrous sulphate

**CORE MODEL**	Score	
**RISK–BENEFIT**	**Mean**	**SD**
**Compared efficacy/effectiveness**	**3.52**	**1.08**
Haemoglobin increase	3.88	1.22
Ferritin/Transferrin saturation	3.74	1.98
Response duration	3.21	1.47
Time until response	4.14	0.90
Health related quality of life (HRQoL)	2.52	2.17
**Compared Safety/Tolerability**	**1.16**	**2.62**
Non-serious and non-fatal adverse events	-0.07	3.17
Serious and non-fatal adverse events	-0.02	2.64
Fatal adverse events	2.81	2.85
Switch of treatment for treatment related adverse event	3.00	1.73
**Patient Preferences**	**1.95**	**2.64**
Preferences on daily frequency of administration	3.36	2.06
Preferences on administration location	0.48	3.53
Preferences on administration procedure	-0.48	3.13
Treatment duration	3.88	2.60
**MODULATORS**		
**Need for intervention**	**2.69**	**2.87**
**Disease Severity**	2.83	3.20
Prepartum-Postpartum consequences of anaemia	3.02	2.92
Ferritin-postpartum depression association	2.60	2.99
**Size of affected population**	2.00	3.87
**Unmet needs**	3.64	1.60
**Type of benefit of intervention**	**2.94**	**1.95**
**Preventive benefit**	2.34	2.45
Peripartum prophylaxis, blood transfusion	2.81	2.57
Prophylaxis, depression	2.26	3.22
Prevention of gestational complications	2.29	2.69
New-born complications	2.14	2.19
**Therapeutic benefit**	3.67	1.45
**Economic consequences of intervention**	**1.06**	**3.32**
**Direct medical costs**	0.28	3.70
Administration cost, healthcare system	-0.40	3.52
Sanitary costs derived from treatment	0.55	3.71
**Direct non-medical costs**	1.64	2.95
**Indirect costs**	1.52	3.29
**Knowledge about intervention**	**2.03**	**2.55**
**Quality of evidence**	2.38	2.48
**Expert consensus/Clinical Practice Guidelines**	2.14	3.08
**Time from treatment commercialization**	1.00	2.83

Scores were obtained in a scale from + 5 (much in favour of ferrous carboxymaltose) to -5 (much in favour of ferrous sulphate)

When deciding the intervention considering the modulator criteria and subcriteria, all of them but one (Administration cost, healthcare system; -0.40 ± 3.52) resulted in favour of FCM, being the most relevant criteria Therapeutic benefit (3.67 ± 1.45) and Unmet needs (3.64 ± 1.60).

### Estimation of the value of intervention and the modulated relative benefit-risk balance

**Fig. 3:**
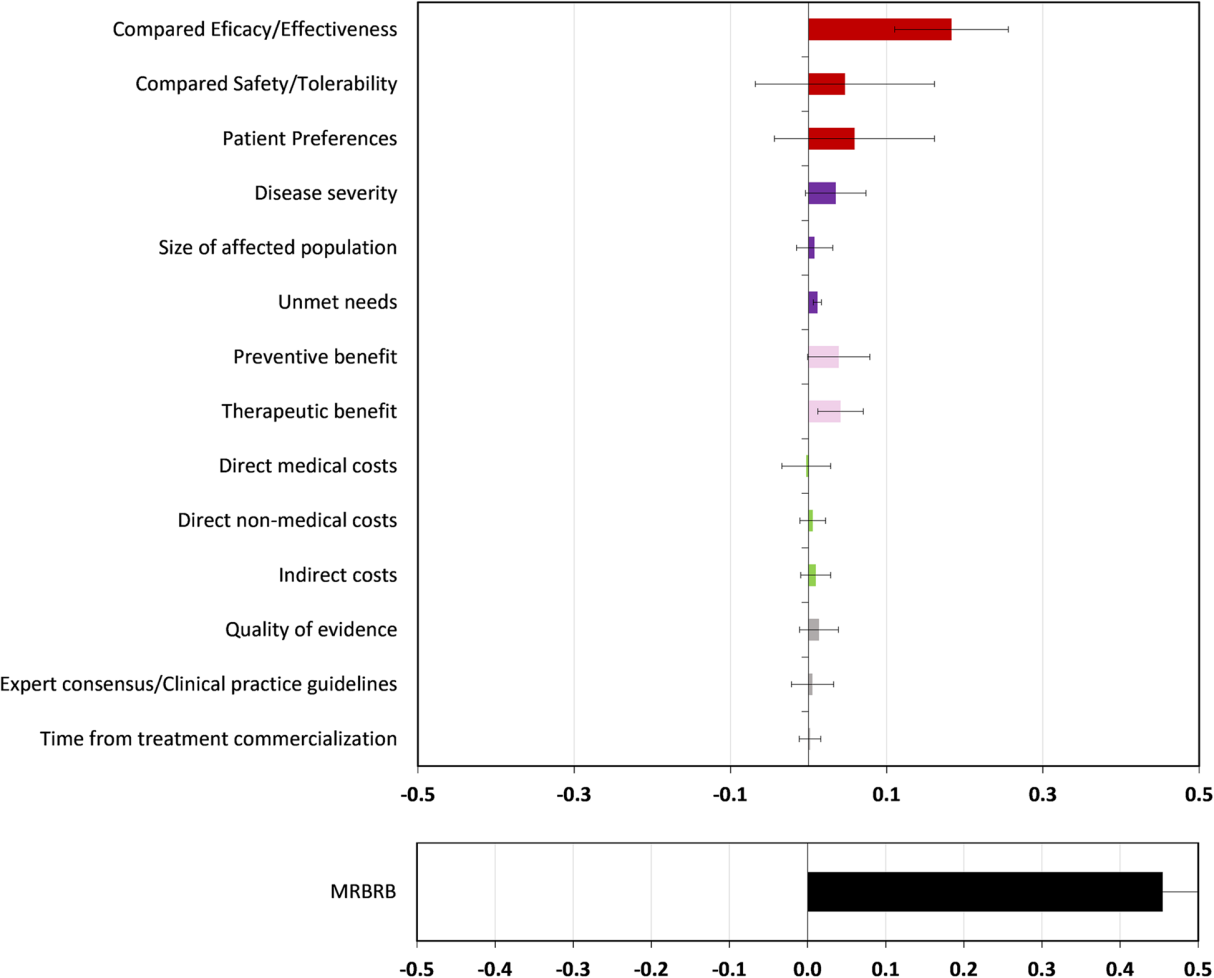
Value of intervention for each criterion of the core model and Modulated Relative Benefit-Risk Balance (MRBRB)

The global MRBRB favoured FCM (0.45 ± 0.1913) in a scale from -1 to + 1 (Fig. 3). Almost all the criteria favoured FCM as well, with values ranging between 0.183 ± 0.07 for Compared efficacy/effectiveness and 0.002 ± 0.01 for Time from treatment commercialization. Only one criterion favoured FS: Direct medical costs (-0.003 ± 0.03).

**Fig. 4:**
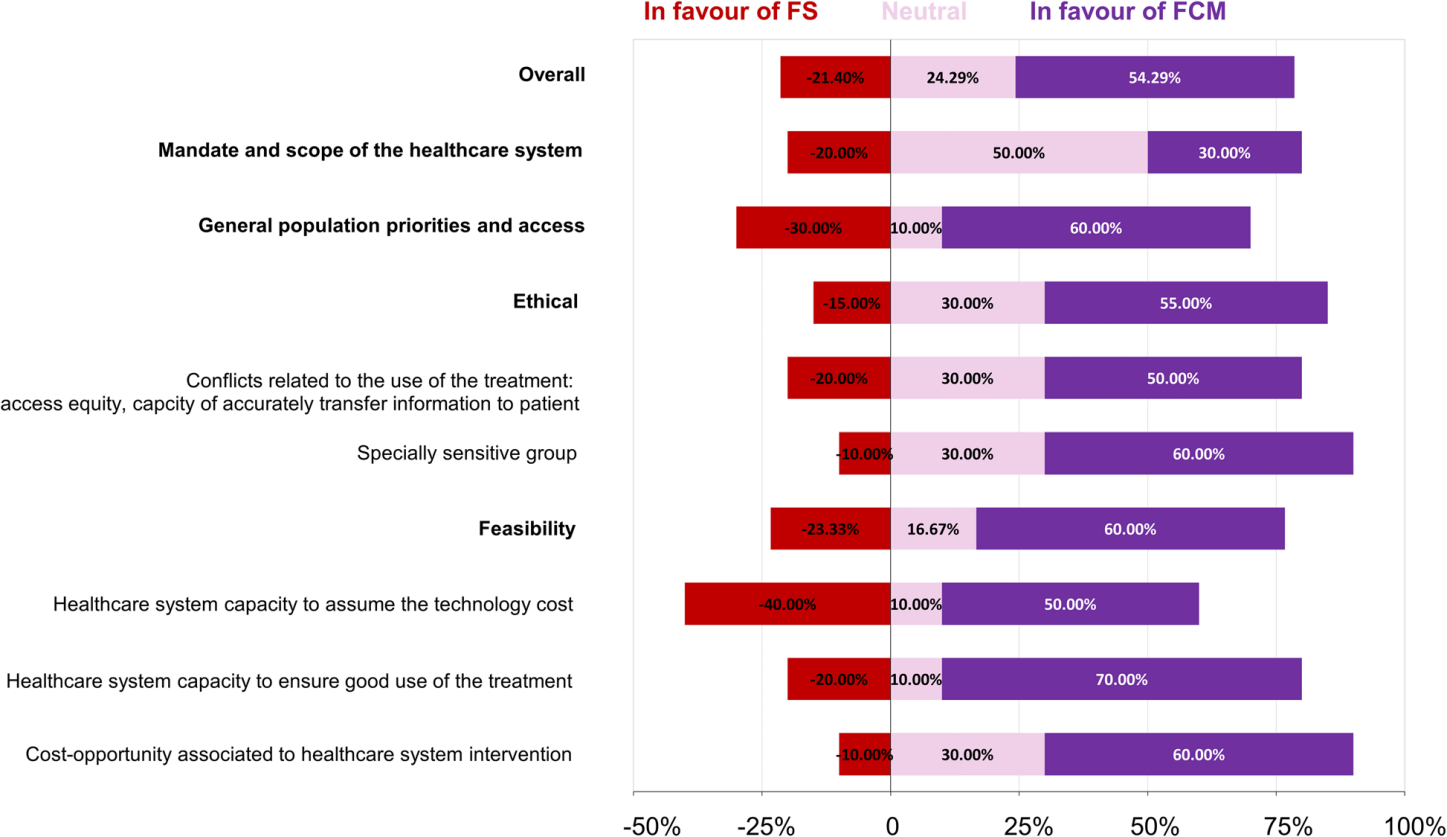
Contextual criteria and percentage of participants considering incorporation of each of the therapeutic alternatives having an impact with respect to each criterion

Regarding the qualitative, contextual criteria, participants scored their preference in terms of in favour of FCM, indifferent, or in favour of FS (without quantitative valuation). In general, evidence presented in most criteria resulted in a positive valuation of FCM (54.29%) (Fig. 4). However, some criteria raised some concern such as: General population priorities and access in which 30% of participants were in favour of FS and 10% were indifferent; and Healthcare system capacity to assume the technology cost, with 40% of participants in favour of FS and 10% not showing any preference.

### Modulated relative benefit-risk balance by profile

From the different perspectives and taking into account the evidence, the decision-making process between FCM and FS was very diverse (Additional Table 3).

Six groups chose FCM regarding risk–benefit data, being the lowest gynaecology/obstetrics (0.11) and the highest anaesthesiology (0.48); while decision-makers chose FS (-0.15). All of them were in favour of FCM according to its Compared Efficacy/Effectiveness (0.07 for decision-makers; 0.12 for gynaecology/obstetrics; 0.17 for patients/patients’ representatives; 0.18 for midwifery; 0.24 for haematology; 0.25 for anaesthesiology, and 0.26 for hospital pharmacy); being the decision mainly driven by the Hb increase. Anaesthesiology was much in favour of FCM regarding Compared Safety/Tolerability (0.27), other profiles chose discretely FCM (midwifery, 0.08; haematology, 0.06; and patients/patients’ representatives, 0.04); hospital pharmacy did not choose any alternative (0.0); and gynaecology/obstetrics and decision-makers were in favour of FS, -0.05 and -0.08, respectively.

Patients’ preferences favoured the decision in favour of FCM by all the participants but decision-makers (-0.14 in favour of FS); with values ranging from 0.04 for hospital pharmacists and gynaecology/obstetrics, and 0.18 for haematology. The only subcriteria generating concerns among those participants in favour of FCM were Preferences on administration procedure.

As for Modulators domain, all participants were in favour of FCM but decision-makers, being especially relevant for this group the Knowledge about intervention (-0.10) and the economic consequences of the intervention (-0.09). The need for intervention was truly relevant for midwifery, anaesthesiology, and haematology; as well as the Type of benefit of the intervention that was also very relevant for these groups and patients/patients’ representatives. Economic consequences of the intervention made three of the seven groups chose FS (hospital pharmacy, gynaecology/obstetrics, and decision-makers, while the rest of the participants were slightly in favour of FCM (0.03–0.09). Regarding Knowledge about intervention, decision-makers were much in favour of FS (-0.10) while anaesthesiology were much in favour of FCM (0.10); the rest of the participants chose FCM with low values (0.01–0.06) or made no choice (0.00 gynaecology/obstetrics).

## Discussion

In the FeminFER project all the relevant stakeholders in gestational and peripartum anaemia assessed the most relevant factors involved in the management of the disease and how these factors affect the decision on the treatment options when deciding between FCM or FS. What must be emphasised about this method is the transparency and systematic data synthesis it offers, and the level of importance of each of the criteria and subcriteria, as has been recently stated in a national report [36], additionally it is a complementary analysis to the classic decision-making process that works as a bridge builder among the different stakeholders involved taking into account many more aspects that should be considered than the classical efficacy, safety, and costs.

All the criteria and subcriteria in the core model (risk–benefit and modulators) were weighted as relevant by clinicians, decision-makers, and patients in the decision-making process of anaemia, which creates a validated framework for anaemia in any other indication, being of special relevance the Compared Efficacy/Effectiveness criteria. Within this domain, the most relevant subcriteria was Haemoglobin increase; however, Ferritin/Transferrin saturation was not considered as being much relevant. This fact brings to light that the real recovery of anaemia, which englobes the restoring of iron stores in the body is not addressed in a fully comprehensive manner by clinicians, who seem to be satisfied by the solely fact of an haemoglobin increase in some cases [13, 14]. Additionally, it has been reported that quality of life is influenced by ferritin levels, which will have a great impact for the patients’ wellbeing [11]. Health-related quality of life was considered the second most important subcriteria, what demonstrates the great implications of this disease in the daily life of patients suffering from anaemia and iron deficit, especially in this sensitive group of women.

Accordingly, within the core-model, the value of intervention with a highest score was Compared Efficacy/effectiveness, followed by Patients’ Preferences, and the last one was Compared Safety/Tolerability. This scoring reflects that, even though all the criteria favoured FCM, Compared Safety/Tolerability was the one raising more concerns among the participants. In relation to the modulator criteria, all but the Economic Consequences of the intervention favoured FCM, this one was scored as in favour of FS by three of twelve of the participating groups.

Regarding the differences observed in the results by profile, these divergences could be explained by the role each of these stakeholders have in relation to the management of the pathology. For example, anaesthesiology and haematology were much more in favour of FCM regarding its compared efficacy/effectiveness profile than other profiles and this is mainly driven by the fact that these clinicians deal with the disease in a late phase, when there is no time for another therapeutic alternative such as in the case of anaesthesiologists or when the most urgent need is to avoid its negative outcomes as has been reported by haematologists. The criterion Compared Safety/Tolerability raised some concerns among the participants, especially the subcriterion Serious and non-fatal adverse events in which three groups chose FS; this fact was discussed by the participants and what was concluded is that those that are less familiar with FCM are more afraid of its adverse events related to its intravenous nature in spite of more than twelve years of clinical experience, however those clinicians that are more used to it state that its benefits clearly overweight its risks. It is of special relevance the score of Need for intervention according to midwives that are one of the professional profiles more involved during pregnancy and postpartum and that have a further knowledge about anaemia beyond future clinical implications in these patients. [7, 28, 37].

Some of the participants were concerned about the economic consequences of the intervention, especially the direct medical costs as the acquisition costs of FCM are much higher than those of FS, however, the general score was slightly in favour of FCM. One of the criteria that made decision-makers lean towards FS was Knowledge about intervention as it can be seen in the value of intervention and stated in the discussion meeting that took place after the decision-making exercise; this is the group that is more used to take decisions based on the available evidence what could influence the scores for these subcriteria as no clear guidelines are yet available for these patients [38]. However, some studies implementing outpatient management with intravenous iron in pregnant women have demonstrated success in the clinical outcomes and are also expected to have an impact in the quality of life of this patients [39].

Overall, the MRBRB favoured FCM over FS for pregnancy and peripartum anaemia in the Spanish context. Furthermore, the developed and validated framework around anaemia allows future integration of newer evidence in order to update the analysis in this indication and also to evaluate the therapeutic alternatives for the management of anaemia in other indications always from an evidence-based perspective.

This work has a number of limitations that need to be discussed, an intrinsic limitation of the methodology is that it allows only the comparison of two interventions, however in our case the most common therapeutic alternatives in the management of pregnant women suffering anaemia in Spain are being compared, being FS the standard of care and FCM one of the first choices when using an IV agent according to clinical experts involved in the MCDA. Additionally, the EVIDEM framework includes several criteria that even though they could apply to most jurisdictions in the EU, the attached weights to the criteria in the framework are highly dependent on the context to which it is adapted, for example economic consequences, unmet needs, or all the contextual criteria, that can be very diverse from one country to another; as well as the decisions made by the participants that rely mainly on the established standard of care, thus translation of our results to other contexts or countries should be carefully evaluated.

## Conclusion

MCDA allows to assess the value of two therapeutic alternatives being compared with a previously validated and common framework and FCM shows a favourable risk–benefit profile, and it is recognised as positively contributing to the management of pregnancy and peripartum anaemia.

## Supplementary Information

**Additional file 1:** **Additional Table 1.** PubMed search strategy. **Additional Table 2.** Cochrane search strategy. **Additional Table 3.** Value of intervention by profile. **Additional Figure 1.** PICO-S-T search strategy. **Additional Figure 2.** PRISMA, Flow diagram of included studies.

## Data Availability

The datasets generated during the current study are not publicly available due to the fact that sessions were held under the Chatham House Rule. This rule implies that “participants are free to use the information received, but neither the identity nor the affiliation of the speaker(s), nor that of any other participant, may be revealed”. However, datasets are available from the corresponding author on reasonable request and in a dissociate manner, with the prior consent of all the participants.

## Abbreviations

FCM: Ferric carboxymaltose
FS: Ferrous sulphate
IDA: Iron deficiency anaemia
IV: Intravenous
MCDA: Multicriteria decision analysis
MRBRB: Modulated relative benefit-risk balance
SLR: Systematic literature review
