# Supplementary material for: Applying reflective multicriteria decision analysis to understand the value of therapeutic alternatives in the management of gestational and peripartum anaemia in Spain
Source: BMC Pregnancy Childbirth. 2022 Feb 25;22:157. doi: 10.1186/s12884-022-04481-w (PMC8881868; doi:10.1186/s12884-022-04481-w)
Supplement: Supplementary file 1 — Additional file 1: Additional Table 1. PubMed search strategy. Additional Table 2. Cochrane search strategy. Additional Table 3. Value of intervention by profile. Additional Figure 1. PICO-S-T search strategy. Additional Figure 2. PRISMA, Flow diagram of included studies. [file 12884_2022_4481_MOESM1_ESM.docx]

Additional Table 1. PubMed search strategy

| PICO-S ELEMENTS | KEYWORDS | SEARCH TERMS | SEARCH STRATEGIES | | |
| --- | --- | --- | --- | --- | --- |
| P | Women suffering gestational and peripartum anaemia | Gestational, peripartum, and Post-partum anaemia | Gestational anemia [MeSH terms]  OR  Gestational anaemia [MeSH terms]  OR  Peripartum anemia [MeSH terms]  OR  Peripartum anaemia [MeSH terms]  OR  Postpartum anemia [MeSH terms]  OR  Postpartum anaemia [MeSH terms] | | |
| I | Ferinject | Iron carboxymaltose-Ferinject | Ferric carboxymaltose  OR  Iron carboxymaltose  OR  Ferinject  OR  Intravenous iron  OR  Injectafer | | |
| C | Ferrous sulfate (Tardyferon®, Ferogradumet®); | Ferrous sulfate, Iron sulfate | Ferrous sulfate  OR  Iron sulfate  OR  Tardyferon  OR  Ferogradumet | | |
| O | Anaemia | Hemoglobin  Transferrin  Ferritine  Reticulocite  Fatigue  Quality of life  Adverse events | Anemia  OR  Anaemia  OR  Hemoglobin  OR  Haemoglobin  OR  Hb  OR  Transferrin  OR  Ferritin  OR  Reticulocyte  OR | Fatigue  OR  Tiredness  OR  Exhaustion  OR  Lack of energy  OR  Weakness  OR  Quality of life  OR  QoL  OR  Adverse events |  |
| S | RCTs  SRs  Meta-analysis  Observational |  |  | | |
| T | Time horizon |  | 2010-Present | | |

Additional Table 2. Cochrane search strategy

| PICO-S ELEMENTS | KEYWORDS | SEARCH TERMS | SEARCH STRATEGIES | |
| --- | --- | --- | --- | --- |
| P | Women suffering gestational and peripartum anaemia | Gestational, peripartum, and Post-partum anaemia | Gestational anemia  OR  Gestational anaemia  OR  Peripartum anemia  OR  Peripartum anaemia  OR  Postpartum anemia  OR  Postpartum anaemia | |
| I | Ferinject | Iron carboxymaltose-Ferinject | Ferric carboxymaltose  OR  Iron carboxymaltose  OR  Ferinject  OR  Intravenous iron  OR  Injectafer | |
| C | Ferrous sulfate (Tardyferon®, Ferogradumet®); | Ferrous sulfate, Iron sulfate | Ferrous sulfate  OR  Iron sulfate  OR  Tardyferon  OR  Ferogradumet | |
| O | Anaemia | Hemoglobin  Transferrin  Ferritine  Reticulocite  Fatigue | Anemia  OR  Anaemia  OR  Hemoglobin  OR  Haemoglobin  OR  Hb  OR  Transferrin  OR  Ferritin | OR  Reticulocyte  OR  Fatigue  OR  Tiredness  OR  Exhaustion  OR  Lack of energy  OR  weakness |
| S | Already limited by database |  |  | |
| T | Time horizon |  | 2010-Present | |

Additional Table 3. Value of intervention by profile.

| ***Value of intervention*** |  |  |  |  |  |  |  |
| --- | --- | --- | --- | --- | --- | --- | --- |
|  | Midwifery | Hospital Pharmacy | Ansthesiology | Gynaecology | Haematology | Decision-makers | Patients/Patients’ representatives |
| **Risk-Benefit** | **0.34** | **0.30** | **0.58** | **0.11** | **0.48** | **-0.15** | **0.36** |
| **Compared efficacy/effectiveness** | **0.18** | **0.26** | **0.25** | **0.12** | **0.24** | **0.07** | **0.17** |
| Haemoglobin increase | 0.08 | 0.12 | 0.08 | 0.04 | 0.05 | 0.02 | 0.03 |
| Ferritin/Transferrin saturation | 0.08 | 0.06 | 0.05 | 0.00 | 0.05 | 0.02 | 0.00 |
| Response duration | 0.03 | 0.02 | 0.03 | 0.02 | 0.05 | 0.00 | 0.01 |
| Time until response | 0.00 | 0.04 | 0.05 | 0.04 | 0.05 | 0.03 | 0.05 |
| Health related quality of life (HRQoL) | 0.00 | 0.02 | 0.03 | 0.02 | 0.05 | 0.00 | 0.08 |
| **Compared Safety/Tolerability** | **0.08** | **0.00** | **0.27** | **-0.05** | **0.06** | **-0.08** | **0.04** |
| Non-serious and non-fatal adverse events | 0.05 | 0.00 | 0.14 | -0.08 | 0.00 | -0.05 | 0.00 |
| Serious and non-fatal adverse events | 0.00 | -0.02 | 0.08 | -0.01 | 0.00 | -0.04 | 0.01 |
| Fatal adverse events | 0.04 | 0.01 | 0.03 | 0.02 | 0.02 | 0.00 | 0.02 |
| Switch of treatment for treatment related adverse event | 0.00 | 0.01 | 0.03 | 0.02 | 0.05 | 0.00 | 0.02 |
| **Patient Preferences** | **0.08** | **0.04** | **0.06** | **0.04** | **0.18** | **-0.14** | **0.15** |
| Preferences on daily frequency of administration | 0.04 | 0.03 | 0.02 | 0.01 | 0.07 | 0.00 | 0.07 |
| Preferences on administration location | 0.00 | 0.00 | 0.02 | 0.00 | 0.05 | -0.06 | 0.00 |
| Preferences on administration procedure | 0.00 | -0.01 | 0.02 | 0.00 | -0.01 | -0.06 | 0.00 |
| Treatment duration | 0.04 | 0.03 | 0.02 | 0.03 | 0.07 | -0.02 | 0.09 |
| **MODULATORS** | **0.39** | **0.08** | **0.35** | **0.05** | **0.35** | **-0.23** | **0.17** |
| **Need for intervention** | **0.12** | **0.05** | **0.10** | **0.02** | **0.10** | **-0.04** | **0.03** |
| **Disease Severity** | **0.08** | **0.02** | **0.05** | **0.02** | **0.08** | **-0.02** | **0.01** |
| Prepartum-Postpartum consequences of anaemia | 0.05 | 0.02 | 0.05 | 0.01 | 0.07 | -0.01 | 0.01 |
| Ferritin-postpartum depression association | 0.03 | 0.00 | 0.01 | 0.00 | 0.01 | -0.01 | 0.00 |
| **Size of affected population** | **0.02** | **0.02** | **0.04** | **-0.01** | **0.00** | **-0.03** | **0.00** |
| **Unmet needs** | **0.01** | **0.01** | **0.01** | **0.00** | **0.02** | **0.01** | **0.01** |
| **Type of benefit of intervention** | **0.20** | **0.03** | **0.09** | **0.04** | **0.10** | **0.00** | **0.10** |
| **Preventive benefit** | **0.1** | **0.003** | **0.056** | **0.014** | **0.07** | **-0.0117** | **0.039** |
| Peripartum prophylaxis, blood transfusion | 0.05 | 0 | 0.0175 | 0.010 | 0.028 | -0.0027 | 0.0164625 |
| Prophylaxis, depression | 0.03 | 0.00 | 0.02 | 0.00 | 0.01 | 0.00 | 0.00 |
| Prevention of gestational complications | 0.01 | 0.00 | 0.01 | 0.00 | 0.02 | -0.01 | 0.02 |
| New-born complications | 0.01 | 0.00 | 0.01 | 0.00 | 0.01 | 0.00 | 0.00 |
| **Therapeutic benefit** | **0.10** | **0.03** | **0.03** | **0.03** | **0.03** | **0.01** | **0.06** |
| **Economic consequences of intervention** | **0.04** | **-0.04** | **0.06** | **-0.01** | **0.09** | **-0.09** | **0.03** |
| **Direct medical costs** | **0.0192** | **-0.027** | **0.024** | **-0.0028** | **0.024** | **-0.06** | **0.002** |
| Administration cost, healthcare system | 0.00 | -0.01 | 0.01 | 0.00 | 0.02 | -0.04 | 0.00 |
| Sanitary costs derived from treatment | 0.02 | -0.02 | 0.01 | 0.00 | 0.00 | -0.02 | 0.00 |
| **Direct non-medical costs** | **0.01** | **0.00** | **0.01** | **0.00** | **0.03** | **-0.02** | **0.01** |
| **Indirect costs** | **0.00** | **-0.01** | **0.02** | **0.00** | **0.04** | **-0.01** | **0.02** |
| **Knowledge about intervention** | **0.03** | **0.04** | **0.10** | **0.00** | **0.06** | **-0.10** | **0.01** |
| **Quality of evidence** | **0.02** | **0.02** | **0.05** | **0.01** | **0.02** | **-0.03** | **0.01** |
| **Expert consensus/Clinical Practice Guidelines** | **0.02** | **0.02** | **0.02** | **-0.01** | **0.04** | **-0.05** | **0.00** |
| **Time from treatment commercialization** | **0.00** | **0.00** | **0.03** | **0.00** | **0.00** | **-0.02** | **0.00** |

Additional Figure 1. PICO-S-T search strategy.

Additional Figure 2. PRISMA, Flow diagram of included studies. ^1^

Records identified through database searching
(PubMed, n =36)

(Cochrane, n=9)

Additional records identified through other sources
(n =32)

Records after duplicates removed and title screening
(n =54)

Full-text articles excluded, with reasons
(n = 11)

Out of scope, n= 5

Type of study, n= 6

Full-text articles assessed for eligibility
(n = 40)

Studies included
(n = 29)

1. Moher D, Liberati A, Tetzlaff J, Altman DG. Preferred Reporting Items for Systematic Reviews and Meta-Analyses: The PRISMA Statement. *PLoS Med*. 2009;6(7):e1000097.
